# Rotational training structures and national employment in public health education: an organizational perspective

**DOI:** 10.1186/s12909-024-06431-w

**Published:** 2024-12-18

**Authors:** Nadieh J. L. M. Taks, Tiuri R. van Rossum, Lidewij T. Warris, Ellen C. Reurslag, Sheda Sadrzadeh

**Affiliations:** 1 Research Group ‘Research & Innovation in Public Health Practice Based Learning’ (RIPPLE), Netherlands School of Public and Occupational Health, 10th Floor, Churchilllaan 11, Utrecht, GV 3527 The Netherlands; 2 School of Health Professions Education (SHE), Maastricht University, Maastricht, The Netherlands

**Keywords:** Postgraduate medical education, Public Health, CFIR, Rotational training structure

## Abstract

**Introduction:**

Postgraduate medical education (PGME) relies on structured training rotations and workplace-based learning (WBL) to provide comprehensive clinical training and professional development. Emphasizing WBL, PGME integrates theoretical knowledge with practical skills through direct patient care involvement, underscoring the pivotal role of training institutes in supporting these initiatives. While curricular changes in PGME have been extensively studied in clinical teaching hospitals, PGME programs in public health (PGME-PH) remain underexplored, yet their multidisciplinary nature post-COVID-19 underscores the urgency for effective curricular reforms. This study explores how training institutions offering PGME-PH navigate the adoption of structured training rotations and a transition to a national employer by examining organizational perspectives to enhance sustainability and integration of educational innovations in this unique context.

**Methods:**

Semistructured interviews were conducted with 14 participants from 8 training institutions offering PGME-PH in the Netherlands between October and November 2022 to explore their experiences with the implementation of a rotational training structure. The interviews were guided by the Consolidated Framework for Implementation Research (CFIR).

**Results:**

The analysis identified three pivotal themes influencing sustainable implementation and ownership: influence, communication, and motivation. Participants expressed concerns about reduced influence in organizing PGME-PH and noted significant communication challenges, such as ambiguity in roles, program frameworks, and financial aspects. Despite these obstacles, participants showed strong motivation for education and underscored the importance of collaboration and shared responsibility.

**Conclusion:**

The successful implementation of structured training rotations was negatively impacted by training institutions’ experience of limited influence and communication challenges. This led to diminished ownership of the new PGME-PH curriculum, potentially affecting its long-term sustainability. Despite these challenges, participants maintain high motivation for educational delivery. Enhancing sustainable implementation requires fostering ownership, promoting collective responsibility, establishing clear communication channels, and nurturing motivation. These factors are crucial for ensuring the success and longevity of educational programs such as PGME-PH.

**Supplementary Information:**

The online version contains supplementary material available at 10.1186/s12909-024-06431-w.

## Background

Postgraduate medical education (PGME) revolves around a structured system of training rotations, in which residents gain practical experience by working in diverse clinical settings and specialties [1]. These rotations form the cornerstone of the PGME framework, offering comprehensive educational experience that is essential for thorough clinical training and professional development [2]. Traditionally, PGME has been grounded in workplace-based learning (WBL), which provides a practical approach to education and assessment through direct involvement in real-life patient care [3]. While training rotations focus on specific clinical domains, WBL encompasses the entire clinical environment as a continuous learning platform, enabling residents to integrate theoretical knowledge with practical skills and develop their competencies in a real-world context. The structured system of training rotations in PGME, which immerses residents in diverse clinical settings and specialties, underscores the pivotal role of training institutions in supporting WBL and rotations. Therefore, engaging these institutions as key stakeholders is essential for successful curricular reform and the advancement of WBL initiatives [4]. This engagement fosters a sense of *ownership* – the degree to which individuals or groups responsible for implementation feel accountable, in control, and committed to an intervention’s success, which is crucial for ensuring sustainable integration and effective implementation of educational innovations [5–8]. High levels of ownership enhance motivation to overcome challenges and ensure the long-term integration of the intervention within an organization or community. The implementation of curricular changes within PGME is a complex process, that is typically examined from the perspective of clinical teaching hospitals [9–11]. However, the perspective of PGME programs in public health (hereinafter: PGME-PH) remains underexplored, despite their distinct educational context and environment. Public health, being multidisciplinary, demands diverse skill sets to address the complex factors influencing health and the efficacy of interventions in populations [12]. Countries worldwide are facing complex and diverse health challenges in the 21st century, such as climate change, humanitarian crises, poverty, gender inequalities and an increasingly complex burden of disease. These challenges increase the urgency for robust public health education, underscoring the importance of effectively implementing curricular reforms in these settings.

To better equip public health physicians for the complexities of future challenges, the Dutch PGME-PH underwent significant curriculum changes in March 2019, with the introduction of a structured rotational training framework. By introducing a rotational clerkship, residents can gain practical experience across diverse public health domains. This approach integrates theoretical knowledge with hands-on skills through WBL, allowing trainees to effectively address public health challenges. In addition, the system of employment of residents changed and transitioned from training institutions to a national employer.

This article aims to explore the process of implementing curricular changes within the PGME-PH, particularly emphasizing the role of the institutions that offer these educational programs. By examining the ownership and responsibility of these institutions, we seek to understand how organizational perspectives, combined with educational and managerial insights, contribute to the successful integration and sustainability of curriculum reforms. We address the following research question: What are the experiences of the training institutions that offer PGME-PH, with a focus on ownership, of the adoption of a structured training rotation framework? Our approach centres on the interplay between organizational strategies and management practices, offering a comprehensive view of how curricular changes can be effectively executed in the workplace learning context. To our knowledge, our study will be the first to investigate this topic within the implementation of innovations in PGME-PH.

## Methods

### Context and participant selection

**Fig. 1:**
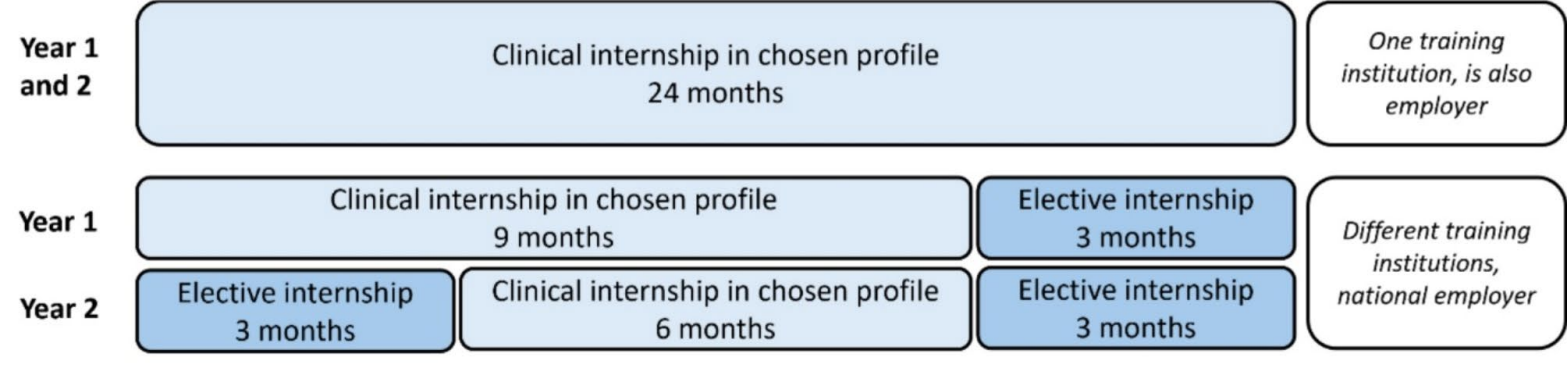
Overview of new curriculum and transfer of employer

Our research context is the PGME-PH in the Netherlands. Residents in the PGME-PH in the Netherlands follow a four year program that consists of two phases of two years: phase one in which a resident is trained in one of eight profiles (e.g. Infectious Disease Prevention and Control or Youth Public Health) and phase two after which a residents becomes a public health physician or specialist with a focus on skills in research, advisement and health policy [13]. In the new curriculum, residents will undertake two clinical internships within their chosen profile, lasting 9 and 6 months. Additionally, they will complete three short elective internships of 3 months each. Previously, residents completed their phase one training at a single institution over two years. The new curriculum was a mandatory change for training institutions nationwide. Figure 1 shows a schematic overview of the original and new curriculum, including employment arrangements.

We made a purposive sample among 25 training institutions, primarily municipal health services. The services are geographically distributed, and vary in size, which is determined by the region and the number of inhabitants. Our sample was selected to capture this diversity. After selection, we asked institutions to identify their most knowledgeable person in the domain of education, which resulted in the inclusion of various formal stakeholder positions such as managers, training coordinators, or (clinical) trainers. Regardless of their specific role, all held a key responsibility in their respective educational programs. Depending on who fulfilled this role, one or two individual(s) were selected per training institution.

### Study theory and design

This study uses the Consolidated Framework for Implementation Research (CFIR), which provides a typology of implementation and is commonly used to assess the implementation of interventions in various settings in the health domain, including PGME [14–17]. The CFIR comprises five major domains and 26 constructs; (1) the intervention, (2) the outer setting, (3) the inner setting, (4) the characteristics of the individuals and (5) the process of implementation. Not all domains and constructs are relevant to a given context.

**Fig. 2:**
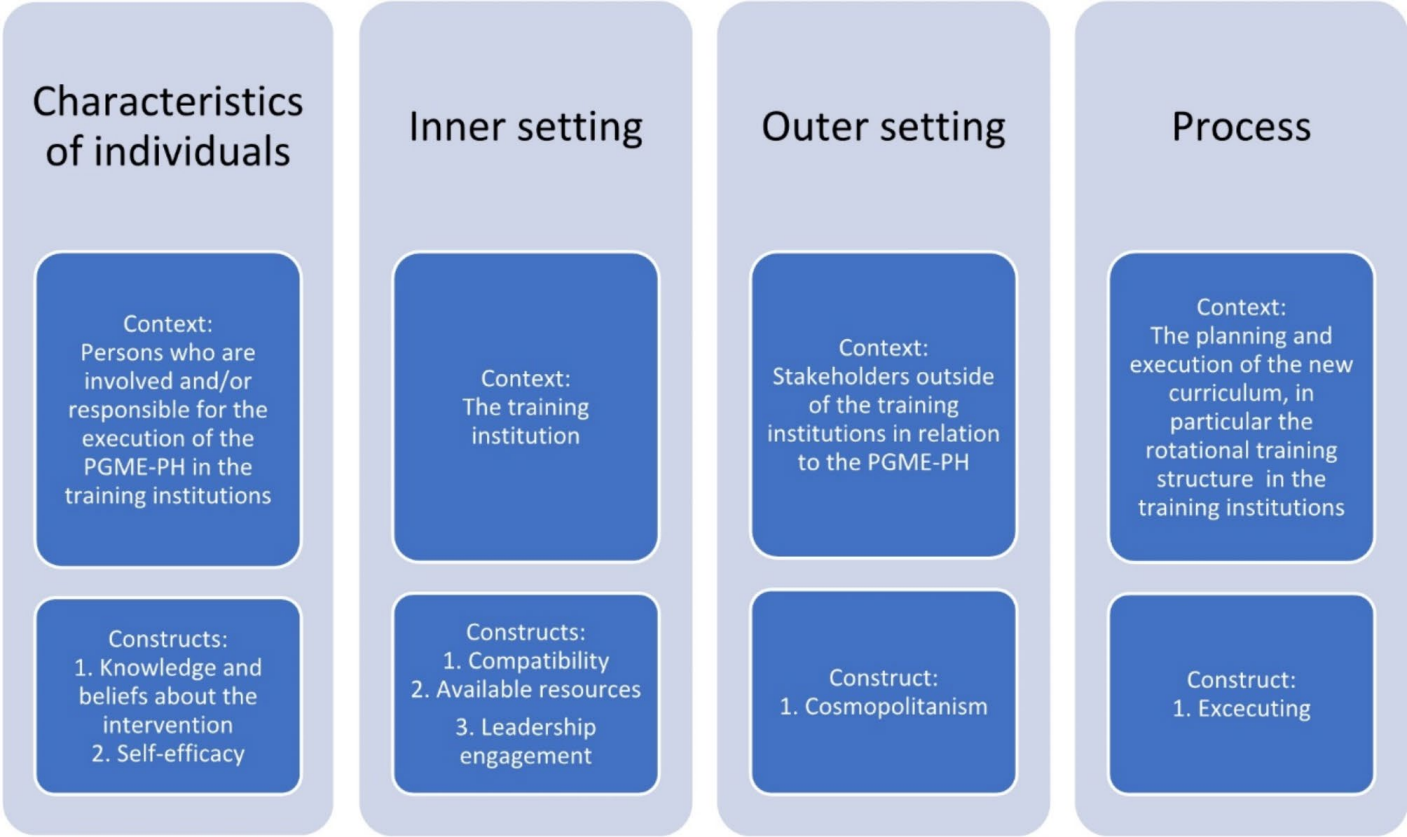
Overview of the used domains and constructs in relation to the research context

We used a qualitative design and data were collected via semi structured interviews. The interviews were guided by CFIR constructs. Based on extensive discussion among members of the research team, we selected four out of the five domains and seven constructs to develop the interview guide, each of which referred to a specific context (Fig. 2). In addition, we selected relevant constructs within these domains. The interview guide was developed for this study and had not been published elsewhere (Appendix I).

The constructs from the intervention domain focus on the different components of an intervention and its adaptability to fit the context. Because the structured training rotation framework is predetermined, we did not include any of the intervention characteristic domain constructs in the interview guide.

The CFIR framework was updated in October 2022 and involved revisions to domains, constructs, and the inclusion, removal, or relocation of constructs. The update occurred after the start of our research and is expected to have no impact on the outcome, as the constructs can be mapped back to the original model to ensure longitudinal consistency [18, 19].

### Data collection

The interview guide, designed by consensus among the research team, was piloted in one interview by two researchers (NT, LW) and adjusted afterwards with one question added and a change in sequence. The interviews were conducted between October and November 2022, either in person or via video conferencing (Microsoft Teams) by NT and LW. Each interview was approximately 60–75 min and was audio-recorded and transcribed verbatim. All participants provided verbal and written consent. Names and other personal data were not transferred to these transcripts. Data collection ended when thematic saturation was reached, defined as the point at which no new codes or themes emerged from the data [20]. This determination was made through ongoing team discussions and careful comparison of emerging themes across interviews. Once it became evident that additional interviews were not contributing any new significant information or unique themes, data collection was concluded.

### Data analysis

The data were analyzed using directed content analysis, a deductive process that starts with an existing theory or framework and utilizes data to either support or build upon that framework [15, 16]. Anonymized transcripts were analyzed by NT and LW, who independently reviewed two transcripts and inductively generated codes that were deductively assigned to chosen constructs in one of the four domains. For this process, we used MaxQDA Analytics Pro 2022©. Discrepancies were reconciled, and a preliminary codebook was created by NT and LW. TvR provided additional insights and interpretations on the codebook. Two more transcripts were coded by NT and LW using the codebook, and refinements were made to finalize it. LW coded the remainder of the transcripts. After completing the final transcript, NT and LW conducted a consensus coding session to discuss the coding process, share their interpretations, clarify the meanings of new codes, and assign codes to the designated constructs and merging codes if necessary. LW then revisited previous interviews with these refined insights. By employing consensus coding, we ensured that the coding process was consistent and reliable, enhancing the dependability of our analysis throughout [21].

The CFIR framework was chosen to guide the analysis from an implementation perspective. Although the interview guide was organized by CFIR constructs, coding was performed independently of the constructs under which the question was organized. The lead author (NT) initially reviewed the coded data, distinguishing various topics and patterns that emerged from the material. Through this inductive coding process, preliminary themes were developed based on recurring ideas and patterns. NT and SS then engaged in a secondary analysis, focusing on identifying deeper, implicit meanings within these patterns. This interpretative approach allowed them to construct three central themes which captured the latent content of the data. In a subsequent meeting NT and SS examined the themes and organized them into a coherent narrative, offering unique insights into the research question.

### Reflexivity

Both NT and LW were residents in public health at the time of conducting the research and followed the new curriculum, which could introduce a positive or negative bias to the analysis. NT conducted the study as an intern of an educational institution that provides lectures during the PGME-PH. Since the educational institution has a close alliance with the training institutions, the involvement of the lead researcher from the educational institution may be perceived as an assessment by the training institution. The same applies to SS, who is a trainer at the educational institution. To mitigate this influence, participants were informed about the roles of NT and SS. TvR, with a background in public administration and a PhD in health professions education, is a trained medical education researcher. ER is also a trainer at the educational institution but did not participate in the interviews. According to previous research, negative comments on the rotational system from an organizational standpoint of the training institutes were expected, which had the potential to introduce a negative bias to the analysis. To manage the influence of these assumptions, the research team frequently reflected on their assumptions, and discussions and decisions were documented in an audit trail that was regularly reviewed.

## Results

We included a total of 14 participants from eight training institutions who were divided into five geographical regions, North (1), East (1), South (1), West (3) and Centre (2). Although all participants held a key responsibility in the educational program, there was much variation in the way participants identified themselves in terms of job titles. In practice, there were no significant differences and they can be summarized into four categories based on responsibilities: training coordinator, trainer, medical/strategic advisor and manager.

The findings were sorted into 3 main themes and related sub-themes: (1) Influence; (2) Communication; and (3) Motivation (Table 1).

**Table 1:** Themes and related sub-themes

Theme	Sub-theme
Influence	- Challenge of continuity - Selection and placement - Loss of ownership
Communication	- Roles and responsibilities - Operational challenges - Shared responsibility
Motivation	- Personal and professional growth - Societal responsibility - Motivation as driving force

## Influence

A common theme voiced by most participants in the study was a perceived lack of influence over the organization and delivery of the PGME-PH program. The theme influence encompassed three sub-themes.

### Challenge of continuity

One of the major concerns raised by participants was the challenge of continuity. In the new rotational system, where residents rotate across multiple institutions over shorter periods, there is limited time for residents to become familiar with the workflows and team dynamics of each institution. In addition, institutions must invest significant time and resources to familiarize residents with workflows and processes. However, this investment often yields limited returns due to the short-term nature of the rotations. This lack of continuity often disrupts team collaboration and patient care.

Well, of course you do notice that someone is here for a short amount of time. I understand that it is interesting to switch, but it also gives you no continuity in your team. That is for the team collaboration as well to the continuity of care. (Manager 4).

However, participants feel that they can influence the practical aspects of the curriculum within their organization, including the number of residents they accept, where to place them, and how to distribute supervision hours among trainers and other staff, factors that are crucial for maintaining continuity of care.

Well, I have a lot of influence on the interpretation in practice, of course. (Training coordinator 5).

### Selection and placement

The lack of influence extends beyond residents’ integration into teams; it also affects the selection and placement of residents. Under the previous system, institutions had greater autonomy in choosing their residents, often selecting individuals already familiar with the organization as they previously had worked there as junior doctors in preparation for their residency. However, in the new system, a national lottery determines where residents are placed, reducing the institution’s influence over who joins their teams.

We notice that the residents who, for example, have already worked for us for a year. If they now are placed with us they already know our organization, they are going through the first nine months much easier. People who come in from the outside for those nine months, well they are sometimes completely overwhelmed. (Training coordinator 1).

### Loss of ownership

The transition from institutional autonomy to a nationalized system has also led to a sense of lost ownership. Training coordinators and managers feel they have limited input into the broader policies and procedural changes imposed from outside their organizations. Many participants expressed uncertainty about where and how they can exert influence within the national structure. While they may have some influence over the practical aspects of the residency within their institution, such as distributing supervision hours and determining resident placements within specific teams, their role in shaping the overall direction of PGME-PH has diminished.

I think in terms of content, we certainly feel ownership. However, in terms of process, we definitely don’t. (Medical/strategic advisor 1).

### Communication

Participants highlighted significant uncertainty and confusion about the overall organization of the PGME-PH, with communication identified as a key barrier. The theme communication identified three subthemes.

#### Roles and responsibilities

A key theme that emerged from the data was the lack of clarity regarding the roles, responsibilities, and accountability of various stakeholders within PGME-PH. Participants expressed confusion about who to approach for support and what their specific responsibilities were within the program. This uncertainty made it difficult for them to navigate the complex network of stakeholders involved.

However, certainly for managers and such, they do not know the network and the turnover is high in terms of managers. It is almost impossible to explain this every time. Therefore, maybe there is a need, especially among managers to know: Hey, what does this stakeholder stand for, who is this stakeholder etc.? A manager sometimes just does not know where should I be? How’s that going? (Medical/strategic advisor 2).

#### Operational challenges

Another key issue highlighted by participants was the lack of effective communication from national stakeholders, for instance regarding the placement of residents, resulting in operational challenges. This led to confusion about training schedules, making it challenging for institutions to coordinate effectively.

Well, what remains complicated is the communication between X, X and X, for example, about training schedules of the residents. We emphasize that you must keep us informed if training schedules change.[] Because we are no longer an employers, we have insufficient insight into this. (Manager 4).

Additionally, financial transparency emerged as a significant concern for many participants, compounding the operational challenges institutions faces. The lack of clarity around subsidies and financial flows left institutions uncertain about funding and costs. As a result, institutions expressed concerns over the sustainability of the PGME-PH program, as financial pressures mounted, straining their ability to provide effective training while maintaining a balanced budget.

We think training is super important; my director also said that we could actually train a lot more residents on the basis of capacity or people who would like to train them. Were it not for the fact that we are running out of money on a financial level. (Training coordinator/trainer 5).

#### Shared responsibility

The third subtheme identified was the need for a shared responsibility. Due to the increased complexity of the new program participants highlighted the importance of shared ownership, where all parties—training institutions, national stakeholders, and residents—take responsibility for the program’s success. Clear communication and the ability to collaborate effectively are essential for promoting this shared responsibility.

It should be well connected and if things do not go well, we all have a role to play. (Training coordinator 3).

#### Motivation

As a last theme motivation was identified as a central driver in the success of the PGME-PH, particularly in the face of challenges like limited influence and communication issues. The theme motivation encompassed three sub-themes.

#### Personal and professional growth

Despite the earlier mentioned obstacles, participants remain deeply committed to the educational mission, driven by a sense of personal and professional growth. Trainers, for example, see the educational process as mutually enriching. They recognize that teaching residents not only benefits the learners but also enhances their own knowledge and expertise, contributing to the growth of both the individual and the institution.

The residents bring us something at an organizational level. However, if you look at it from the perspective of the trainer he/she says: “I have a resident, he/she enriches me, I enrich that resident. [] I train this resident, this person to be a beautiful doctor, and transfer that profession onto them”. (Medical/strategic advisor 1).

#### Societal responsibility

Beyond personal fulfillment, participants emphasized the broader societal responsibility tied to education. Many expressed the importance of ensuring that the next generation of professionals is adequately trained to continue the work of the field. This sense of shared responsibility reinforces their dedication to the success of PGME-PH, with trainers seeing themselves as integral to sustaining the profession.

If you want your profession to continue to exist, you also have to ensure that new people arrive, and you also have to train them. You have to contribute to that. That is also a certain social responsibility that we all share. (Training coordinator 5).

#### Motivation as a driving force

Participants emphasized that success and continuity of the program relies on passionate and dedicated individuals who take pride in their roles, as without them, the education would not exist. While the new curriculum offers benefits like increased learning opportunities for residents through the rotational system, the pressure of shorter training periods can strain motivation, making the need for passionate individuals even more critical.

I think that has been the added value and the saving grace of the residency. So those passionate people you truly need them, you truly need them a lot. In addition, you have to keep that connection with each other, to feel that ownership and to want to go for it. (Manager 3).

## Discussion

In this study, we explored the experiences related to adopting a structured training rotation framework and transferring to a national employer in the PGME-PH. Our focus was specifically on ownership, using the CFIR framework as our guide. Our analysis revealed three major themes that appear to interfere with ownership: influence, communication and motivation. Below, we first discuss our themes and how they relate to existing literature on effective implementation, particularly in terms of ownership and sustainability. Next, we offer recommendations for practice.

In recent years, there has been growing interest in exploring the implementation of educational innovations in healthcare organizations, yet the sustainability of these initiatives has received comparatively little attention [6, 22]. This lack of sustainability leads to the failure of many promising initiatives and the absence of long-term benefits [7]. Insight into facilitators and barriers to sustainable interventions, such as ownership, are therefore invaluable.

The theme *Influence* reflects participants’ struggle with a diminished sense of control over resident training, particularly concerning selection and placement procedures and operational aspects. This perceived loss of influence can be understood as a shift from control over both the content and process of training to primarily content alone. While institutions retain control over the implementation, the centralized nature of resident selection and placement reduces their control over staffing and planning. This disconnection affects institutions’ sense of ownership, making them feel disengaged from the overall program and can have several ramifications. First, it can lead to decreased commitment toward the new PGME-PH framework. When stakeholders feel that their input holds less weight, their investment in the program’s success may dwindle. Second, it can foster a sense of disengagement and potentially hinder the integration and sustainability of the new curriculum. This finding aligns with the existing literature on educational change, which emphasizes the importance of stakeholder buy-in and a sense of agency in fostering successful implementation [8, 15, 16, 23]. To promote long-term sustainability and foster innovation, institutions should have greater influence over operational aspects of the program, such as placement decisions and training schedules. This increased control would enable them to better align educational goals with institutional needs, strengthening their sense of ownership. This could involve adjusting the application process and developing an online planning tool to optimize training schedules and improve staffing. These measures can bridge the ownership gap and enhance sustainability. Next, participants faced limited *Communication* and ambiguity surrounding organizational responsibilities and stakeholder roles. Role ambiguity and lack of clarity are known to hinder sustainability [6, 8, 17]. This lack of communication can influence attitudes within training institutions, affecting their sense of ownership and commitment to sustainability. Furthermore, due to limited communication, the passive approach taken in adopting complex innovations rather than an active, proactive stance was noted. This “let it happen” approach contrasts with the effective “make it happen” strategy needed to support sustainable implementation [24, 25]. To address this, clear communication channels should be established and training institutions should implement regular, structured meetings with clear agendas involving all key stakeholders. These meetings should focus on clarifying organizational responsibilities, discussing the placement and progress of residents, and ensuring alignment between training objectives and operational needs. By fostering transparent and proactive communication, institutions can improve stakeholder buy-in, reinforce a sense of ownership, and support the sustainable implementation of the PGME-PH framework. Finally, we observed a strong *Motivation* for education among individuals involved in the PGME-PH, particularly among trainers, despite limited influence and communication issues. Breckenridge’s theory of Motivating Change highlights that lasting change depends on favorable psychosocial-structural conditions, which include individual motivation, collective support, and effective management of resources and systems [26]. The success of the PGME-PH relies heavily on dedicated participants being passionate about training and education. However, maintaining this equilibrium becomes precarious without the right collective support (i.e. such as communication issues), and effective management (i.e., lack of influence). Therefore, ensuring transparent communication and active stakeholder engagement is vital for sustaining motivation, promoting ownership, and ensuring the long-term viability of the PGME-PH framework. In addition to motivation, training institutions demonstrate a strong commitment to education, often prioritizing resident training over their core business operations. Larger services are better equipped to handle uncertainties, while smaller services express scepticism about the sustainability of the current structure. A key challenge for these institutions is balancing the investment in training with the productivity and contributions of residents. This concern highlights the need for improved support in optimizing resident deployment, ensuring that institutions can benefit from residents’ contributions while managing the financial and operational costs of training. One example could be developing a uniform onboarding document that accelerates integration, and ensures that residents can contribute more effectively from the start. This will create a healthier work environment that balances investing in residents with reaping the benefits.

### Limitations

This study has a relatively small sample size from multiple municipality health services. Some services could not participate due to a lack of residents, limiting the generalizability of the findings. However, our confidence in their validity is strengthened by their alignment with previous internal evaluations and discussions of the educational institute (i.e., the NSPOH). Additionally, the study employed interview questions and coding processes guided by a theoretical framework, effectively capturing crucial aspects of the implementation experience.

## Conclusion

In conclusion, training institutions remain motivated to facilitate and execute the PGME-PH which is driven by their responsibility to pass on their profession and expertise, and their social role in educating the next generation. However, the implementation of the rotational structure and transfer to a national employer has transformed their once leading role into a facilitating role. The lack of influence and uncertainty, particularly in communication, creates a delicate balance, and any further disruption raises concerns about the sustainability of the current PGME-PH. This is illustrated by the following quote: *‘We’re all losers at the moment.’ (Training coordinator 3)*.

## Electronic supplementary material

Below is the link to the electronic supplementary material.

Supplementary Material 1

## Data Availability

The datasets used and/or analyzed during the current study are available from the corresponding author on reasonable request.
