# Supplementary material for: Rotational training structures and national employment in public health education: an organizational perspective
Source: BMC Med Educ. 2024 Dec 18;24:1434. doi: 10.1186/s12909-024-06431-w (PMC11654320; doi:10.1186/s12909-024-06431-w)
Supplement: Supplementary file 1 — Supplementary Material 1 [file 12909_2024_6431_MOESM1_ESM.docx]

| **Selected domains based on Damschroder et al. 2009^[[1]](#footnote-1)^:** | **Selected constructs** | **Definition** | **Related interview questions** |
| --- | --- | --- | --- |
| **Characteristics of individuals**  *Context: Persons who are involved and/or responsible for the execution of the PGME-PH in the training institutions.* | 1. Knowledge & Beliefs about the intervention | Individuals’ attitudes toward and value placed on the intervention as well as familiarity with facts, truths, and principles related to the intervention. | 1. How do you/does your organization experience the implementation of the rotational training structure?  - Are you encountering any issues? If so, how do you address them?  2. What influence do you have over the training program?  - How does that affect you?  - Is that sufficient? |
|  | 2. Self-efficacy | Individual belief in their own capabilities to execute courses of action to achieve implementation goals. | 1. Do you/does your organization feel equipped to execute the training program?  - Why or why not?  - How does that affect you in terms of your role and function? |
| **Inner setting**  *Context: The training institution.* | 1. Compatibility | The degree of tangible fit between meaning and values attached to the intervention by involved individuals, how those align with individuals’ own norms, values, and perceived risks and needs, and how the intervention fits with existing workflows and systems. | 1. How does the rotational structure affect existing work processes and colleagues on the work floor?  - On paper, only the duration of the residents’ internship has been adjusted. This should not cause any problems in practice. What do you think about that? |
|  | 2. Available resources | The level of resources dedicated for implementation and on-going operations, including money, training, education, physical space, and time. | 1. To what extent are you/your organization facilitated by the organization in the implementation of the training program?  - How does that affect you in terms of your role and function? What do you need? |
|  | 3. Leadership engagement | Commitment, involvement, and accountability of leaders and managers with the implementation. | 1. To what extent do you/does your organization experience support and involvement from the organization in the implementation of the training program?  - How does that affect you, and is it enough? |
| **Outer setting** *Context: Stakeholders outside of the training institutions in relation to the PGME-PH.* | 1. Cosmopolitanism | The degree to which an organization is networked with other external organizations. | 1. To what extent do you feel ownership over the rotational training structure?  2. How does it affect the organization that residents are no longer employed by you? |
| **Process**  *Context: The planning and execution of the new curriculum, in particular the rotational training structure in the training institutions.* | 1. Executing | Carrying out or accomplishing the implementation according to plan. | 1. What is your role within the organization, and what are your responsibilities concerning the training program? |

**Appendix I: Interview guide
*Consolidated Framework for Implementation Research***

**Interview guide

Research Question**What are the experiences of the training institutions that offer PGME-PH, with a focus on ownership, of the adoption of a structured training rotation framework?

**Introduction**I would like to discuss your experiences with the implementation of the rotational training structure and transition to a national employer.

1. What is your role within the organization, and what are your responsibilities concerning the training program?
2. How do you/does your organization experience the implementation of the rotational training structure?
   - Are you encountering any issues? If so, how do you address them?
3. How does it affect the organization that residents are no longer employed by you?
4. What influence do you have over the training program?
   - How does that affect you?
   - Is that sufficient?
5. To what extent do you feel ownership over the rotational training structure?
6. To what extent are you/your organization facilitated by the organization in the implementation of the training program?
   - How does that affect you in terms of your role and function? What do you need?
7. To what extent do you/does your organization experience support and involvement from the organization in the implementation of the training program?
   - How does that affect you, and is it enough?

In the next section, I would like to discuss how the rotational training structure fits within your organization.

1. How does the rotation structure affect existing work processes and colleagues on the work floor?
   - On paper, only the duration of the residents’ internship has been adjusted. This should not cause any problems in practice. What do you think about that?
2. Do you/does your organization feel equipped to execute the training program?
   - Why or why not?
   - How does that affect you in terms of your role and function?

**Closing**Are there any points we haven’t discussed yet?

**Probing questions**

- How do you deal with this?
- How does that affect you/your organization?

1. Damschroder, L.J., et al., *Fostering implementation of health services research findings into practice: a consolidated framework for advancing implementation science.* Implement Sci, 2009. **4**: p. 50. [↑](#footnote-ref-1)
